# Comprehensive Analysis of Chemical Structures That Have Been Tested as CFTR Activating Substances in a Publicly Available Database CandActCFTR

**DOI:** 10.3389/fphar.2021.689205

**Published:** 2021-12-08

**Authors:** Manuel Manfred Nietert, Liza Vinhoven, Florian Auer, Sylvia Hafkemeyer, Frauke Stanke

**Affiliations:** ^1^ Department of Medical Bioinformatics, University Medical Center Göttingen, Göttingen, Germany; ^2^ CIDAS Campus Institute Data Science, Georg-August-University, Göttingen, Germany; ^3^ Institute for Informatics, University of Augsburg, Augsburg, Germany; ^4^ Mukoviszidose Institut gGmbH, Bonn, Germany; ^5^ German Center for Lung Research (DZL), Partner Site BREATH, Hannover, Germany; ^6^ Clinic for Pediatric Pneumology, Allergology, and Neonatology, Hannover Medical School, Hannover, Germany

**Keywords:** cystic fibrosis, substance database, compound database, therapeutic substances, high-throughput screening, library collection, chemical space annotation, search tool

## Abstract

**Background:** Cystic fibrosis (CF) is a genetic disease caused by mutations in *CFTR*, which encodes a chloride and bicarbonate transporter expressed in exocrine epithelia throughout the body. Recently, some therapeutics became available that directly target dysfunctional CFTR, yet research for more effective substances is ongoing. The database CandActCFTR aims to provide detailed and comprehensive information on candidate therapeutics for the activation of CFTR-mediated ion conductance aiding systems-biology approaches to identify substances that will synergistically activate CFTR-mediated ion conductance based on published data.

**Results:** Until 10/2020, we derived data from 108 publications on 3,109 CFTR-relevant substances via the literature database PubMed and further 666 substances via ChEMBL; only 19 substances were shared between these sources. One hundred and forty-five molecules do not have a corresponding entry in PubChem or ChemSpider, which indicates that there currently is no single comprehensive database on chemical substances in the public domain. Apart from basic data on all compounds, we have visualized the chemical space derived from their chemical descriptors via a principal component analysis annotated for CFTR-relevant biological categories. Our online query tools enable the search for most similar compounds and provide the relevant annotations in a structured way. The integration of the KNIME software environment in the back-end facilitates a fast and user-friendly maintenance of the provided data sets and a quick extension with new functionalities, e.g., new analysis routines. CandActBase automatically integrates information from other online sources, such as synonyms from PubChem and provides links to other resources like ChEMBL or the source publications.

**Conclusion:** CandActCFTR aims to establish a database model of candidate cystic fibrosis therapeutics for the activation of CFTR-mediated ion conductance to merge data from publicly available sources. Using CandActBase, our strategy to represent data from several internet resources in a merged and organized form can also be applied to other use cases. For substances tested as CFTR activating compounds, the search function allows users to check if a specific compound or a closely related substance was already tested in the CF field. The acquired information on tested substances will assist in the identification of the most promising candidates for future therapeutics.

## Introduction

Cystic fibrosis (CF) is a genetic disease inherited in an autosomal recessive fashion (Elborn, 2016). The highest incidence is observed among people with northern European ancestry where it affects approximately one out of 3,000 newborns in populations who offer CF genetic testing to couples (SpringerMedizin, 2021). The disease-causing gene *CFTR* encodes a chloride and bicarbonate transporter expressed in exocrine epithelia throughout the body (Elborn, 2016). Manifestations of the generalized exocrinopathy encompass failure to thrive and recurrent pulmonary infections as the hallmarks of the two major affected organ systems, i.e., the gastrointestinal and the respiratory tracts (Elborn, 2016).

The clinical diagnosis of CF is assisted by bioassays that rely on the detection of CFTR dysfunction in the sweat gland (Elborn, 2016), in the nasal and in the intestinal epithelium (Wilschanski et al., 2016). Symptomatic therapy at centers specialized in CF care has increased the life span of CF patients considerably: while CF was once known as a devastating disease leading to death in infancy or early school age, the average survival of CF patients is now by 40 years in developed countries (Elborn, 2016). Hallmarks of therapy improvement were the supplementation of pancreatic enzymes and a consistent treatment of infections of the respiratory systems (Elborn, 2016). For a few years, therapeutics are available that directly target dysfunctional CFTR, developed for clinical application by Vertex Pharmaceuticals (Van Goor et al., 2006). While Kalydeco targets the CFTR mutant G551D-CFTR, Orkambi is licensed for the most frequent CFTR disease causing lesion F508del-CFTR (Martiniano et al., 2016). Surprisingly, the experience with these causal treatments that complement the successful symptomatic treatment falls behind the enthusiastic expectations leading to “efforts from the community to look for other therapeutics in spite of Orkambi” (Martiniano et al., 2016). The Cochrane Reviews conclude in summary that “Combination therapies (lumacaftor–ivacaftor and tezacaftor–ivacaftor) each result in similarly small improvements in clinical outcomes in people with CF; specifically, in improvements in quality of life (moderate-quality evidence), in respiratory function (high-quality evidence) and lower pulmonary exacerbation rates (moderate-quality evidence) (Southern et al., 2018). Taken together, CFTR mutation-specific therapeutics became available, but research for more effective substances is ongoing (Gentzsch and Mall, 2018).

The database CandActCFTR aims to provide detailed and comprehensive information on candidate therapeutics for the activation of CFTR-mediated ion conductance using a systems-biology approach to identify substances that will activate CFTR-mediated ion conductance in a synergistic fashion based on published data. There are several efforts to identify compounds that activate CFTR residual function in the community: Hit-CF Europe specializes to identify compounds that can be used to treat rare CF mutations in an organoid model (HIT-CF, 2021). Recently, Veit et al. (2018) and Phuan et al. (2018) have combined therapeutics derived from their screening efforts. In addition, several competing pharmaceutical companies develop CFTR therapeutics through their own screening data (CFF, 2021). Our approach differs from all of these efforts as it is not restricted to a particular CFTR mutation type and not restricted to a particular screening data set. In contrast, we have merged publicly available information in a meta-database enabling comprehensive data retrieval and analysis. To the best of our knowledge, our effort is the only holistic approach to use integrated data from multiple sources employing advanced digital technologies to provide unbiased criteria for selecting therapeutic substances. This strategy should be particularly suitable to successfully select substance combinations as several of the compounds published by academia showed small effects, albeit they were tested in many bioassays. This is in contrast to the high-throughput-screening strategy as here, effective compounds are selected based on one assay only. Interestingly, Veit et al. (2018) could recently show that a combination of compounds that perform poorly when considered isolated will improve mutant CFTR function to 50 or 100% of wild-type level, confirming that all substances that activate CFTR might be valuable therapeutics.

## Materials and Methods

The CandActCFTR project is organized into three project domains:

• The data sources regarding CFTR and ways to search and access these.
• Means to afterwards handle and organize the data coming from these sources.
• Data extracted, annotated, and stored for structured access.

### Data Sources and Ways to Search and Access These

To facilitate that the developed workflows are adaptable by other research groups for other use cases, we focused on using open-source resources and modularized our system as well as we could. For the project, we collected a pool of literature derived data regarding chemical compounds in the context of cystic fibrosis, especially focusing on interactions with the CFTR protein. The aim was to facilitate the collection, organization, and thereafter user-friendly representation of the collected data to a research community. To achieve this, we mined the PubMed service (PubMed, 2021) to find a list of relevant entries to inspect. We organized this shared literature list using the free to use Zotero web service (Zotero, 2021) and documented our search at https://www.zotero.org/groups/1632179/candactcftr_public_references. We then used Zotero’s application programming interface (API) to pull the citation data into our information system.

The chemical structure is the root of our data model and all other information is linked directly or indirectly with this root. We provide an entry form to enter structures in the database, either by simply providing identifiers or drawing the molecular graph. The system queries PubChem (Kim et al., 2021) with the provided identifiers for more information, for instance synonyms. Users can draw their molecule online using the Ketcher JavaScript plugin (Ketcher, 2021) and then translate this graph to an isomeric SMILES, which is used to look up the compound after being converted to an InChIKey (Southan, 2013) using OpenBabel (Open Babel, 2021) by the server. Alternatively, if the user provides a PubChem ID (Kim et al., 2021), this is used to collect the isomeric smiles and InChIKey (Southan, 2013) and synonyms into our database. In case of a match, the system can create links to the PubChem’s web resources (Kim et al., 2021) for this compound, when shown in the web views. If no match is found querying PubChem (Kim et al., 2021) via InChIKey (Southan, 2013), the compound is stored without the PubChem (Kim et al., 2021) synonyms and saved annotated with just the name entered by the user. InChIKeys (Southan, 2013) are used to look up structures and prevent double entries.

### Means to Handle and Organize the Data

To achieve our aim, we needed a classical server stack with storage (domain/databases), request & data handling (Controllers), and visualizing interfaces (views). The data system should be easily extendable and accessible by multiple means of loading and querying the data. Data table definitions should be adaptable without extensive training. Therefore, we chose the groovy-dialect-based Java implementation of a Grails (Grails Framework, 2021) system as our base for the project (https://candactcftr.ams.med.uni-goettingen.de/). The models/domain definition can be done using simple and structured human-readable text documents containing the declarations of the data variables to be stored and the interconnection of these tables, while the details to instantiate and update the actual data tables in the used database system of choice is taken care of by the Grails (Grails Framework, 2021) system itself. After the instantiation of the data structures within the database server of choice, one can either use the Grails (Grails Framework, 2021) controller system to query the data resources and ultimately display some of the data or process the data and display the results. Because the back-end database system in the Grails (Grails Framework, 2021) stack can be a generic Structured Query Language (SQL) server, any means to access and manipulate the data tables using APIs can be used, thus allowing the use of established data analysis pipeline tools like KNIME (Berthold et al., 2007; KNIME, 2021) to interface the data. Administrative tasks like backups can thus be handled by known tools like mysqldump (MariaDB KnowledgeBase, 2021) and phpMyAdmin (phpMyAdmin, 2021) or others, depending on the used backend system for storage. For small projects with few tables, KNIME (Berthold et al., 2007) can be used and later extended for batch updating data tables. To enable the storage of the literature data in a way compatible to a common exchange format as provided by Zotero (2021), we used the Citation Style Language schema of the Citation Style Language (CSL) project (Citation Style Language, 2021) to generate a generic data representation for literature meta-data (a domain model) to be used in our Grails (Grails Framework, 2021) web server. This definition is not CFTR specific and can thus be used in other projects. In our implementation, Grails Framework (2021) creates from this abstract definition a representation in the attached database MariaDB (MariaDB Foundation, 2021). Grails Framework (2021) has connectors for multiple target database systems and can be adapted to local infrastructure prerequisites. It contains supports of in-memory databases, thus reducing minimal installation requirements to Java and facilitates the start of the development phase as well as deployment. As data sets can be shipped as text files with the Grails Framework (2021) implementation to be loaded on startup of Grails Framework (2021), this is an easy way to provide a preset yet interactive view for the data tables or web APIs.

### Data Extracted, Annotated, and Stored for Structured Access

Literature references are internally stored in the CSL format. Similarly to the entering of compounds, we integrate references with their meta-data via the PubMed API (Kim et al., 2021) querying their PubMed ID and receiving titles, authors and journal. After compounds and literature references have been entered, we provide web interfaces to help with linking compounds to literature. This web interface provides the remote working curator with the information of what has been linked to one substance before, and allows the curator to select a new reference guided by a search form-based workflow to pick a reference from the literature list. To allow easier batch processing however, likely done by curators who have direct working access to the backend of the database, we also provide similar maintenance workflow pipelines again using KNIME. We integrated so far three ways of providing molecular graph images to the web views: dynamic integration of a PubChem (Kim et al., 2021) derived Portable Network Graphics (PNG), by accessing PubChem’s Web API, or independent of an internet connection using the Kekule JavaScript libraries (Jiang et al., 2016) in our web view pages to render the graph directly on the client machine, as our third option we now create the PNGs on the server side using Open Babel (2021).

In many instances in CandActCFTR, KNIME (Berthold et al., 2007) is used as a back-end controller and analysis pipeline. The literature search was enhanced by automatization: we used the PubMed (Kim et al., 2021) API via search nodes from KNIME (Kim et al., 2021) to perform further searches, improving our literature references by automatic annotations with data not stored previously. We also used the PubChem (Kim et al., 2021) API to convert large lists of compound names found in some paper supplements without structure information, to extend the structure data set, defining molecular compounds by drawing molecular graphs.

While interactive web pages can provide easy access to search perspectives of common interest, deeper analysis or rapid prototyping requires a more flexible way of interacting with the stored data not only in our system but also when cross-linking to other resources. Since we cannot implement all potential use-cases, but as we suggest that this tool is used for other applications, we provide access to the raw data tables that are accessed via pipeline tools like KNIME (Berthold et al., 2007) to allow rapid building of a prototype workflow for specific aspects to be analyzed within the data and to motivate to generate a specific web view. An example of how this might look like can be seen on https://candactcftr.ams.med.uni-goettingen.de/Compound/showFullSetChemSpaceInfluenceOnCFTRFunction, where we use the open source ECharts-JavaScript library ECharts first developed by Baidu (Li et al., 2018a) and now the Apache Foundation (Apache ECharts, 2021) to load a JavaScript Object Notation (JSON) formatted data set on the client side to depict our chemical space as an interactive scatterplot with CFTR annotations and links to the landing pages of the respective compounds, where one can investigate further annotations such as references.

### Modeling a KNIME Workflow to Retrieve and Extend Spreadsheet-Defined Content

**FIGURE 1:**
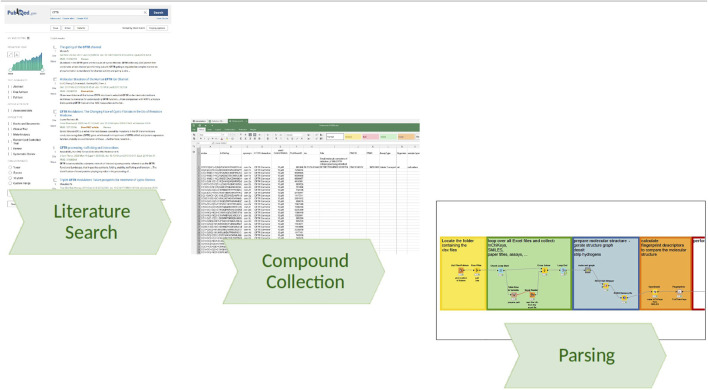
Information flow from literature search to database. Beginning with a literature search (e.g., via PubMed), we collect the PDFs in a structured folder system and convert the information into a tabular format, adhering to a common minimal column requirement (e.g., SMILES, PubMedID, CFTR Interaction), allowing for any other relevant column definition, and finally parsing the file stack using KNIME pipelines, consolidating the tables, extending the information with annotations from other sources such as synonyms from PubChem, and calculating molecular descriptors for a subsequent similarity search.

Starting with a list of papers extracted from a literature search (e.g., PubMed (Kim et al., 2021) search term “CFTR”), one is left with the task of organizing how to first get the papers and then extract the information. At first, we do not know what type of data we might extract (e.g., pictures, supplements, sketches), and it might differ very much between individual papers as a source. Thus, it is advantageous to have a folder with individual subfolders to collect the data for each paper, ideally named so that we can identify it quickly (e.g., first author and year). The goal is to copy the paper into the respective folder and then start a spreadsheet to summarize the excerpts (e.g., compound identifiers, assay). One can create and fill these folders automatically using KNIME (Berthold et al., 2007) and an input list of references coming from the citation manager, programming it to cycle through the list and creating the folders with names simply concatenated from first author, year, and maybe PubMed (PubMed, 2021) ID (PMID). Either automatically or if something similar was already done by hand, one can import folder names to guide later analysis and quality checking by annotating data found within the folders. Using this system allows to retrace where the data came from and in case of missing or faulty data one can go back to one particular folder and source spreadsheet file. In other words, this method requires accepting the use of spreadsheets as the first step in the processing of data but enables an improvement of quality by iterating over its content. Going from the unstructured list of papers mainly in PDF format, obtained from the initial PubMed search, sorting the data into such an organized folder structure is providing first means to organize the data further and the resulting data stack can be seen as a pseudo-database, which can be parsed by computational approaches and transformed further to answer specific question (see also Figure 1).

A KNIME (Berthold et al., 2007) pipeline looks for all xlsx files it encounters in a specified folder location and will also cycle through any subfolders it encounters within. After the list is defined, a loop will load all files and concatenate the columns where possible, additionally the path of the information is accessed, which can be broken down for folder and file names. To behave as a means to collect chemical information like structures, we defined a minimum column to be “smiles” (Weininger, 1988), which should contain an isomeric SMILES representation of the molecule “(C1=CC(=CC=C1C2=COC3=CC(=CC(=C3C2=O)O)O)O)”; for quality control the InChIKey (Southan, 2013), if given in the source, is recommended, as the SMILES encoded structure can be deterministically converted to an InChIKey. Another identifier is the PCID (PubChem Compound Identifier) used at the PubChem repository. For defining the literature source it came from, we recommend, if possible, to use the PubMed (Kim et al., 2021) ID (PMID) as we can extract all other information (title, authors, doi, journal, …) from PubMed (2021). These general information columns, which hold data that are valid for each entry in the table collecting the information retrieved by the pipeline during the loop, have to be filled only in the first row and the pipeline takes care of extending this information to each row, after the import of the xlsx file. All additional columns will be collected and attached one after the other, with columns with the same name occurring in multiple files being merged into one column. When the pipeline is executed again, it updates the mapping of the columns and joins the information. Analogously, for errors uncovered, one always has the option to alter the pipeline structure in KNIME to fix this in the workflow or in the source data. The benefit is that the current state of the data at execution time is preserved and one can then export the collected data in various formats or upload the data to the server instance.

### Interfacing With PubChem-API

To realize CandActCFTR, we have used KNIME (Berthold et al., 2007) to interface the PubChem (Kim et al., 2021) service, the PubMed (PubMed, 2021) service, and chemical tool kits such as OpenBabel (2021). We use multiple services of the PubChem (Kim et al., 2021) web service to amend our data sets, where possible. Straight forward, to resolve structures of entries with reported PubChem Identifier (PCID), the linked information on the PubChem (Kim et al., 2021) servers, such as the isomeric SMILES (Weininger, 1988) and InChIKey (Southan, 2013; InChI Trust, 2021), can be pulled for entries that did not yet specify them yet. *Vice versa*, check if we can resolve an InChIKey (InChI Trust, 2021) to a PubChem (Kim et al., 2021) entry, for linking our entry to this additional source. In both use cases, we can then query for synonyms of the compound if a PCID exists, thus the initial list that is checked for coverage in PubChem is amended by a synonyms list queried from this service. This list might be used for further direct matching with other data sources (e.g., papers, patents), which only contain names for the compounds but no chemical information.

### Interfacing With PubMed-API

We also used the PubMed (PubMed, 2021) web services to query for additional information to amend our data set where having a PMID to directly get the meta-data for a specific literature resource and in batch mode for multiple entries at once. If only information like the “title” is known, we can use this to inquire PubMed (PubMed, 2021) for potential matches and in case of a defined match amend the meta-data. KNIME (Berthold et al., 2007) can be extended with multiple open-source chemistry-related tool kits (Chemistry Development Kit, 2021; Open Babel, 2021; RDKit, 2021). Thus, after importing a structure containing column from a data source, these toolkits are used to convert the molecules into different formats, for instance, to export Structure Data Format (SDF) files or create molecular descriptors for which CandActCFTR uses RDKit (2021).

### KNIME-Based Similarity Search

The similarity search provided by our webserver is encapsulated in another KNIME workflow and uses a list of SMILES as input, which are then combined with the data stored in the SQL database. As KNIME enables the storage of data within a workflow, we can provide our preloaded data set as a reference to be used also in offline environments, or keeping specific archived versions. We are using the PubChem fingerprints computable by the chemoinformatics nodes in KNIME for similarity search/calculating distances. For the creation of the PCA coordinates, we use the available standard descriptors (e.g., MW, logP) in KNIME and use the correlation filter to reduce the dimensionality removing redundant information before calculating the PCA. The PCA serves mainly to help visualize the chemical space for the online site and serves to get a first glance of potential overlap regions. In the future, we might pass the descriptors through as well to be selected by the user and enable 3D views like with our similarity search results.

### Web Resources

The CandActCFTR webpage is provided at: https://candactcftr.ams.med.uni-goettingen.de/.

The software, the CFTR data content, and documentation are provided at: https://gitlab.gwdg.de/mnieter1/CandActBase.

## Results

**FIGURE 2:**
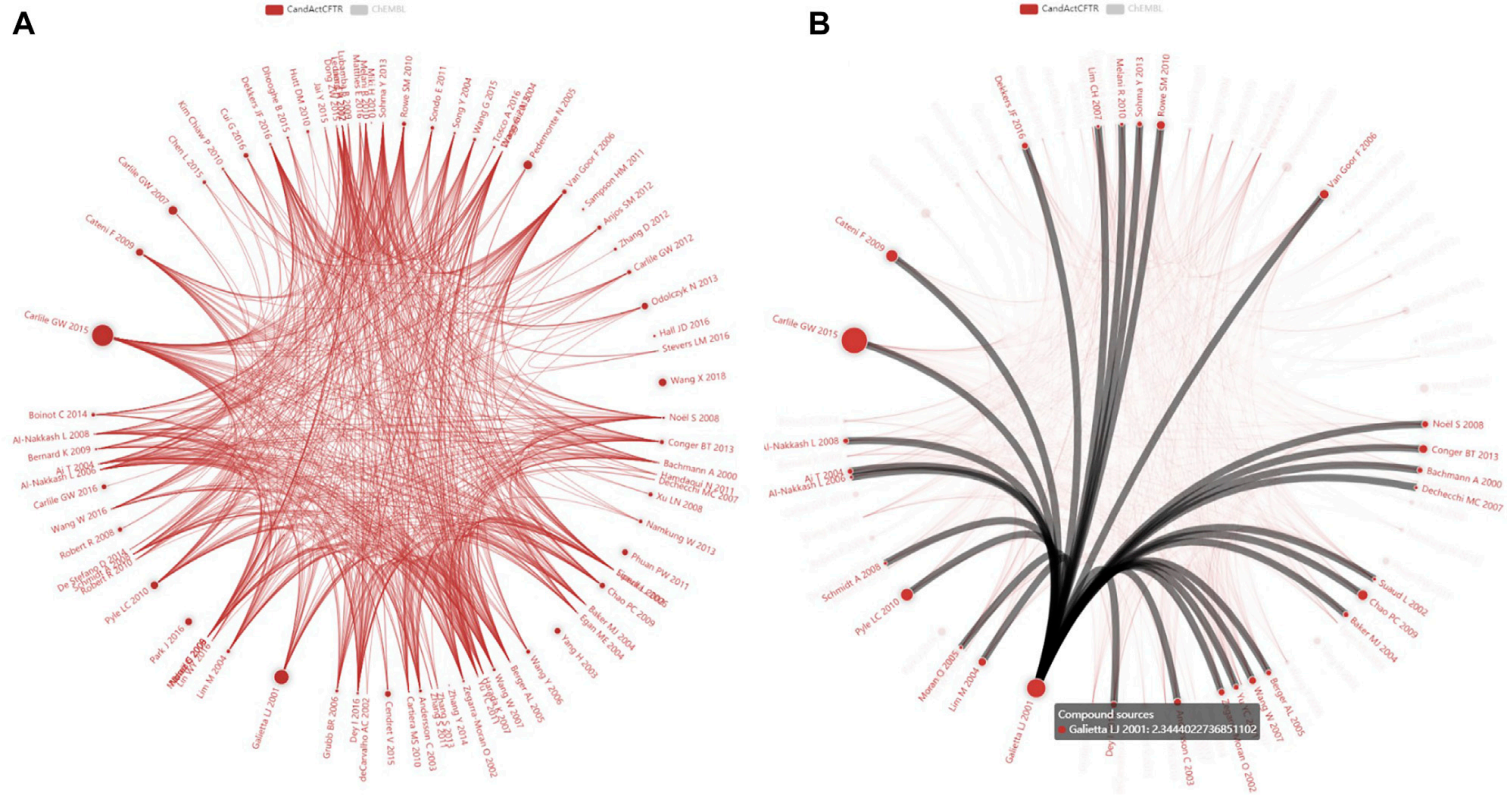
Impact of individual publications on CandActCFTR content. The screenshots **(A, B)** in the diagram show each publication as a circle whereby the number of substances is visualized as the circle diameter (Log10 scaled). Connections between contributions describe substances that are listed in more than one publication. **(A)** represents the actual CandActCFTR publication dataset, hiding the also loaded ChEMBL reference data set and is displayed on our website at http://candactcftr.ams.med.uni-goettingen.de/Compound/showFullSetChemSpaceCompoundOverlaps BetweenPublications where it is provided with a zoom function, and **(B)** shows additional information on links and on publications upon mouse-over of connections or circles, e.g., all publications highlighted with at least one overlap compound to Galietta et al. (2001). Interestingly, a contribution with a large content but only few shared substances exist (e.g., Pedemonte et al., 2005) and some authors describe a set of unique substances (e.g., Miki et al., 2010; Phuan et al., 2011; Sampson et al., 2011; Hall et al., 2016). Below this diagram on the webpage are tables summarizing the interactions with individual overlap counts, providing also links to the publications.

Within the last 3 years, we have collected data on molecules tested as CF therapeutics from several resources, screening the literature and chemical databases for substances that are reported to activate CFTR residual chloride secretion or elevate the expression of CFTR and compiled this information in a database. Until 10/2020, we could derive data from 108 publications (Figure 2) on 3,109 CFTR-relevant substances via the literature database PubMed (PubMed, 2021) and further 666 substances via ChEMBL (Gaulton et al., 2012) whereby only 19 substances were shared between these sources. This poor overlap was expected as databases such as ChEMBL, DrugBank (Wishart et al., 2018), and others recruit their content from different sources and thus share only a minority of structures (Southan et al., 2013). Strategies realized by the researchers to uncover CFTR therapeutic substances were 1) direct testing of a therapeutic substance that resembles an already known CFTR activating substance structurally (example: flavonoids) or influences a pathway that is known to be vital for CFTR (example: inhibitors of autophagy) or 2) high-throughput screening of a chemical substances bank with a CFTR-relevant assay.

Our database, described in detail in methods, is assembled from the following principle modules: The software framework uses open-source resources and integrates existing tools and resources, which allows CandActCFTR to be repurposed for adaptation to other use cases. The software is based on Grails Framework (2021) for which content is provided by the established data analysis pipeline tool KNIME (Berthold et al., 2007). PubMed (2021) was mined to find a list of relevant entries to further inspect, which were organized as a shared literature list using the Zotero web service (Zotero, 2021). Literature meta-data were incorporated into our system through the Citation Style Language schema of the Citation Style Language (CSL) project (Citation Style Language, 2021). The chemical structure is the root of our data model and all other information is linked directly or indirectly with this root. Direct incorporation of chemical structures was facilitated by the Ketcher (Ketcher, 2021) JavaScript plugin that provides the means to draw molecular structures and translates these graphs to isomeric SMILES (Weininger, 1988). CandActCFTR next converts isomeric SMILES to an InChIKey (Southan, 2013; InChI Trust, 2021) using OpenBabel (2021). Additional information about chemical structures were retrieved from information resources such as the shared literature and converted to database content by providing identifiers or drawing the molecular graph. Synonyms, isomeric SMILES, and InChIKey (Southan, 2013) are retrieved from PubChem’s Web API (Kim et al., 2021). Molecular graph images on CandActCFTR were first provided by accessing PubChem’s (Kim et al., 2021) Web API or using the Kekule JavaScript libraries, but are now replaced by pre-generated images using OpenBabels “SMILES to PNG” function. Processed CandActCFTR content for the web page is provided, for instance, through the open source ECharts-JavaScript library (eCharts Baidu (Li et al., 2018a) is now part of the Apache Foundation (Apache ECharts, 2021)), which receives a JSON formatted data set, provided by Grails (Grails Framework, 2021), on the client side, to depict our chemical space as an interactive scatterplot with CFTR annotations.

Data on CandActCFTR substances are provided on our webpage at https://candactcftr.ams.med.uni-goettingen.de/. The ordering principle is the chemical structure of the compound, which we archive by its isomeric SMILES (Weininger, 1988) and its corresponding InChIKey (InChI Trust, 2021). We use the InChIKey (InChI Trust, 2021) as a unique identifier to retrieve corresponding entries from PubChem (Kim et al., 2021), and add links to those resources. Generic names and all available used synonyms are provided with each compound. Compounds are affiliated with all publications in which the compound is mentioned, and the key message of the publication is provided to the reader as a short reference-into-function (RIF) text. Apart from information about the project, we provide

**FIGURE 3:**
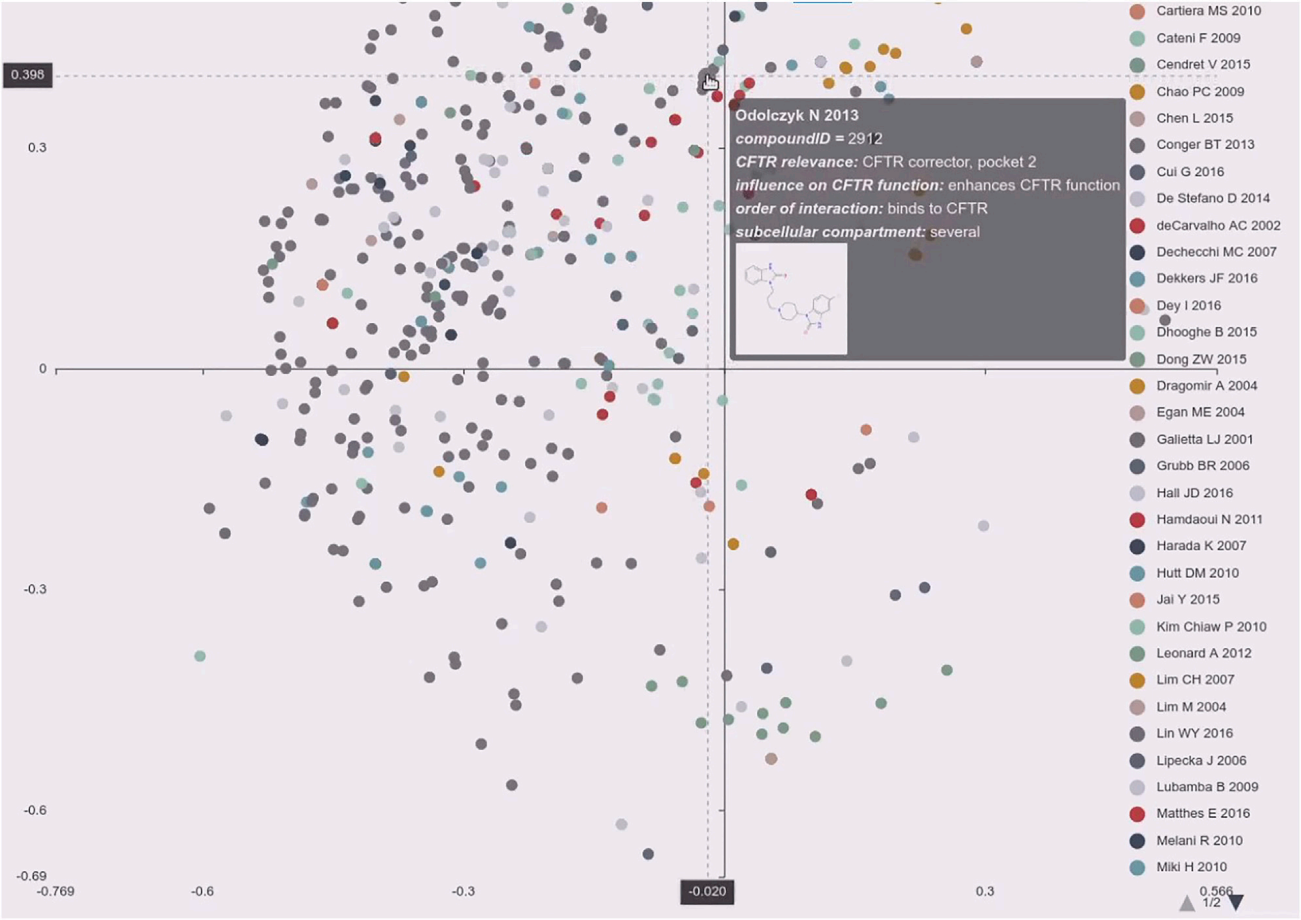
Exploring the distribution of individual publications over the chemical space. The screenshot shows the principal component analysis plot of the chemical descriptors labeled by publication. The plot is interactive and using the legend the users can select/deselect certain publications. It supports zooming and upon mouse-over on a data point the quick summary is displayed consisting of the paper references short name, compound id, main annotations like *CFTR relevance*, *influence on CFTR function*, *order of interaction*, *subcellular compartment*, and the molecules graph. Actual clicking on the dot will load the specific compound page in a new tab in the browser (https://candactcftr.ams.med.uni-goettingen.de/Compound/showFullSetChemSpacePaperAssociationSortedBySize).

**FIGURE 4:**
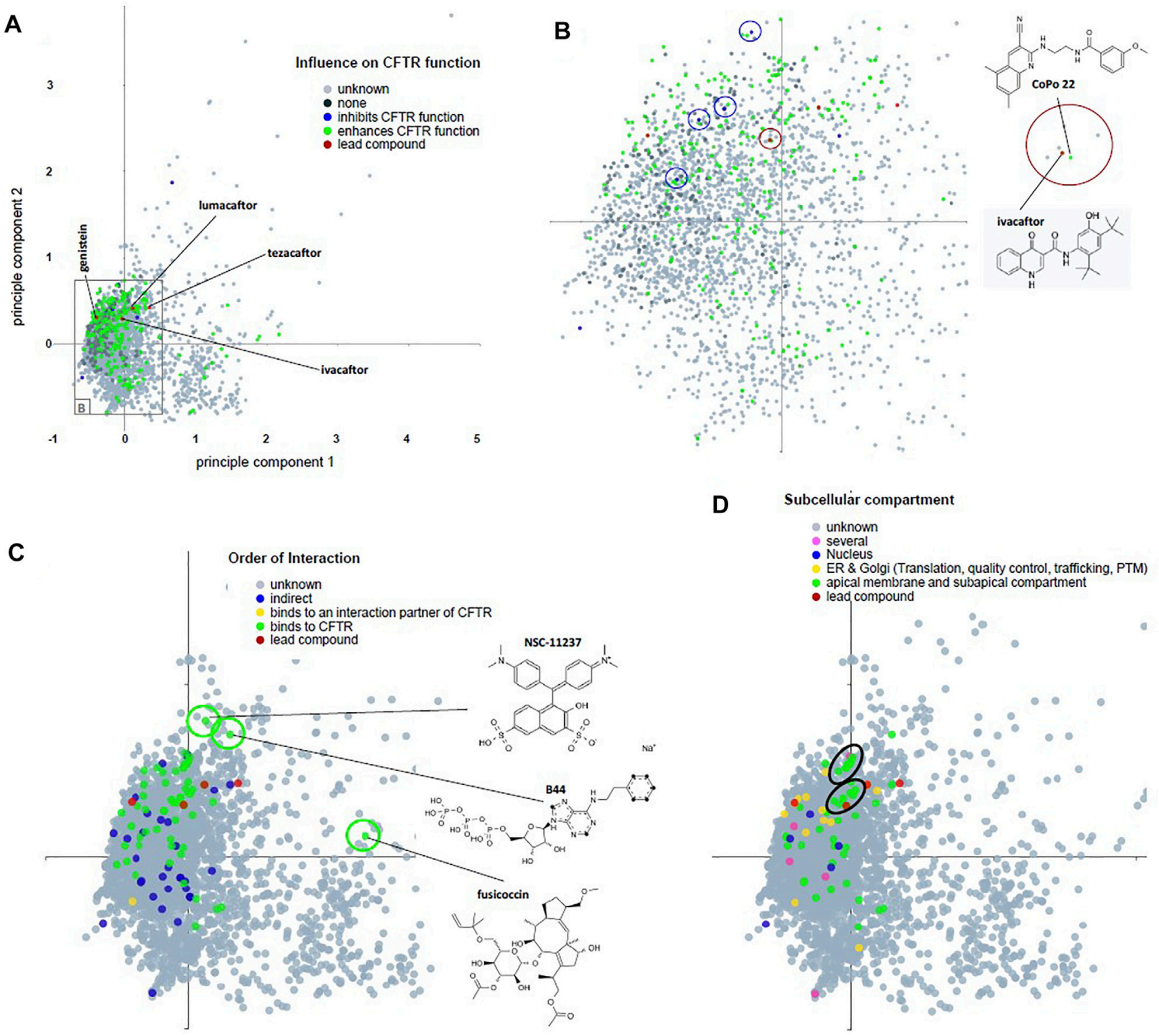
Principal component analysis of descriptors for chemical attributes of CandActCFTR compounds and their classification in three categories. **(A–D)** Genistein and the Vertex molecules lumacaftor, ivacaftor, and tezacaftor are represented by red symbols (labeled in **A**) as lead compounds. **(A)** Dataset annotated for influence on CFTR function (see Table 1). Inconsistent assignment and unknown are summarized as one category (gray). Compounds that enhance CFTR function occupy the central space of the PC diagram whereby their occurrence roughly reflects the density of compounds tested. **(B)** Compound-rich central area as indicated in **(A)**. We are aware that the current PC analysis projects a multidimensional space into two dimensions; as a consequence, compounds in close proximity are unlikely to share all attributes. However, we have noticed that CFTR inhibitors and CFTR activators are closely located (blue circles) and that the lead compound ivacaftor has a nearby neighbor CoPo22 (InChI Trust, 2021). **(C)** Compounds stratified by order of interaction. Three structural outlier compounds are marked by a green circle, indicating an area of interest in the chemical space in a region where few tested compounds are within our database. Outliers are fusicoccin (Chemistry Development Kit, 2021), the non-hydrolyzable ATP analogon B44 (RDKit, 2021), and NSC-11237 (Gaulton et al., 2012). **(D)** Compounds stratified by subcellular compartment. Two clusters of substances that act at the apical membrane in the subapical compartment are encircled in black. Interactive PC diagrams are provided at https://candactcftr.ams.med.uni-goettingen.de/Compound/index.

**FIGURE 5:**
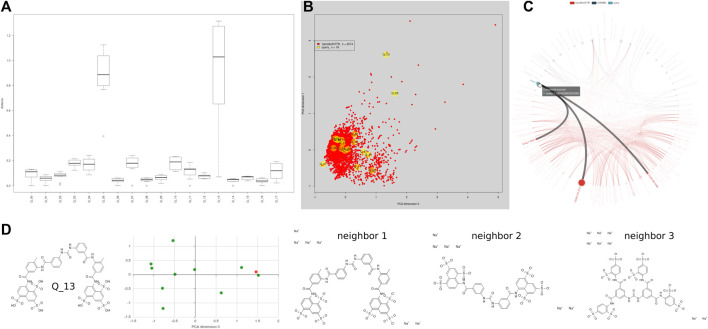
Using the similarity search to investigate the overlap of new data sets of interest with the annotated chemical space, for example, a small ENaC data set. Forty-six entries from 18 different publications about ENaC, resulting in a set of 18 distinct structures, some with multiple entries distributed over publications, e.g., 11× amiloride. The resulting set was used for a similarity search using the form @ https://candactcftr.ams.med.uni-goettingen.de/Compound/searchCompounds. **(A)** Analysis identified six direct overlaps and shows the relative distance of the closest 10 compounds, **(B)** 16 out of 18 query structures cover generally the same area as the existing CandActCFTR point cloud and two rather distinct compounds can be seen, but one entry has a single close neighbor in CandActCFTR space. **(C)** Three listed papers contain an overlap, where Carlile et al. (2015), Grubb et al. (2006), and Song et al. (2004) all contain amiloride; while Carlile et al. (2015) also contained exact matches for benzamil, phenamil, LY-294002, pioglitazone, and rosiglitazone. **(D)** Using the individual structure similarity search, one can have a more distinct look at the structures surrounding the query structure; for example, looking at the query Q_14 suramin and its 10 closest neighbors, we can see that the closest neighbor is actually the query structure with another ionization state. Q_05 Vasopressin is a cyclic polypeptide and has no close by neighbors, but its assigned closest neighbor is another cyclic polypeptide called tyrothricin.

• A site that allows searching for compounds by names, SMILES (Weininger, 1988), or InChIKey (InChI Trust, 2021). Using structural information encoded in SMILES format, we also provide the means for similarity search, also for lists of compounds. Hereby, novel structures can also be drawn into a window and directly converted to SMILES (Weininger, 1988) using Ketcher (Ketcher, 2021) (see https://candactcftr.ams.med.uni-goettingen.de/Compound/searchCompounds and Figure 5 and Supplementary Videos S2–S4).
• Information/data on the individual compound summary pages containing chemical structure information, synonyms, affiliated publications with explanatory RIF text, classification information for its influence on CFTR, its order of interaction with CFTR, and the cellular compartment in which it works. As the InChIKey is a universal identifier also used on many other sites, we linked a Google search using the InChIKey for convenience to identify other resources, e.g., potential vendors (see https://candactcftr.ams.med.uni-goettingen.de/Compound/cycleCompounds/1).
• Information/data on all compounds: an interactive depiction of the chemical space derived from their chemical descriptors via a principal component analysis (see, e.g., https://candactcftr.ams.med.uni-goettingen.de/Compound/showFullSetChemSpaceInfluenceOnCFTRFunction and Figures 3, 4), showing as popups on mouse-over on a data point also the structure and main annotations like *CFTR relevance*, *Influence on CFTR function*, *Order of interaction*, and *Subcellular compartment*. Upon mouse-click, the corresponding compound page is loaded.
• Information/data on all publications: an overview of relative size and overlaps between publications is provided, also including the ChEMBL-derived reference data set as connection graph and table view (see https://candactcftr.ams.med.uni-goettingen.de/Compound/showFullSetChemSpaceCompoundOverlapsBetweenPublications or alternatively using the chemical space depiction again but colored by paper association instead https://candactcftr.ams.med.uni-goettingen.de/Compound/showFullSetChemSpacePaperAssociationSortedBySize).

See also the short supplement video captures of using the webservice:

• Search_Ivacaftor_By_Name.mp4
• Search_Ivacaftor_By_Drawing_Exact_Structure.mp4
• Search_Ivacaftor_By_Drawing_Structure_One_MethylGroup_Off.mp4 - implicitely activating similarity search
• Exploring_PointCloud_TaggedByPaper_with_CompoundImagesAndAnnotationPopUps.mp4

The following examples may serve to illustrate that the effort undertaken by collecting data from different resources and through the joint analysis of these data has already revealed valuable insights into how CFTR-acting substances can be identified and understood:

1. Among the 3,109 substances listed in CandActCFTR, 145 molecules do not have a corresponding entry in PubChem (Kim et al., 2021) or ChemSpider (2021), which indicates that there currently is no single comprehensive database on chemical substances in the public domain.
2. We now have an overview of systematic screens for compounds undertaken for cystic fibrosis in academia. Verkman, University of California, San Francisco (Galietta et al., 2001; Yang et al., 2003; Pedemonte et al., 2005; Phuan et al., 2011; Namkung et al., 2013); Galietta, Genova, Italy (Galietta et al., 2001; Pedemonte et al., 2005; Cateni et al., 2009; Pedemonte et al., 2011); and Hanrahan & Thomas, McGill University, Montreal, Canada (Carlile et al., 2007; Robert et al., 2008; Carlile et al., 2012) contribute most data in that regard and dominate the field; in addition, Xu et al. have screened Chinese medicinal herbs for substances that act on CFTR (Xu et al., 2008). Moreover, Cui et al. (2016) have used a pharmacophore modeling approach to predict CFTR activating substances.
3. Ibuprofen and glafenine are both identified as CFTR-activating substances (Robert et al., 2010; Carlile et al., 2015). Both are nonsteroidal anti-inflammatory drugs (NSAIDs). It is interesting to note that these two NSAIDs both target and partially correct the CF-typical, proinflammatory status as genes that determine immunology and inflammation, having been uncovered as CF modifying genes, target the basic defect of impaired ion conductance in cystic fibrosis epithelia as well (Stanke et al., 2011). In other words, the two NSAIDs suggested as CFTR activating substances and the identified CF modifying genes both emphasize the weight of the inflammatory pathway for the manifestation of the CF basic defect.
4. Some substances have been tested extensively in several biosystems by different groups. For instance, resveratrol has been published as a CFTR activating compound in five cell lines and primary cells by CFTR protein visualization, by CFTR function in transepithelial current measurements in primary cells as well as animal tissues and by patch clamp in two heterologous expression systems and *in vivo* in a mouse model (Hamdaoui et al., 2011; Zhang et al., 2013; Zhang et al., 2014; Dhooghe et al., 2015; Jai et al., 2015). Even though these bioassays appear to cover the entire spectrum of CFTR-relevant assays, no conclusion is reached by the field on the application of resveratrol as a therapeutic agent as two research groups conclude that resveratrol does not work or even inhibits CFTR.
5. A similar controversy is seen for miglustat, tested in 10 different bioassays encompassing cell lines and data from mouse models (Norez et al., 2006; Lubamba et al., 2009; Norez et al., 2009; Jenkins and Glenn, 2013; Leonard et al., 2013; Europe PMC, 2021; Noël, 2021). A follow-up investigation on whether or not the substance activates CFTR in nasal epithelium in CF patients reports no effect. However, recently it was noticed that even approved drug Orkambi does not improve NPD in all cases (Graeber et al., 2018), and moreover, that the correction of the basic defect by Orkambi did not predict the influence of Orkambi on the clinical parameters assessed by Graeber and colleagues, suggesting that a weak performance of the substance miglustat might not be contradictory to the primary data obtained in the preclinical phase.

We conclude from our survey that even if a substance has been confirmed as a CFTR activating agent in several bioassays, the field does not rely on these data for choosing such a substance as a chemical scaffold for future drug development. Thus, relevant information can be overlooked. While CandActCFTR collects data from CFTR-related screens, its structure can easily be adapted to other data collections. As an example, we here provide our second use case for ENaC-activating substances, and show the similarity search for overlaps of CFTR-tested compounds with ENaC-tested compounds, depicted in Figure 5. The query contains suramin, which is also present in our CandActCFTR data set, derived from Carlile et al. (2015), where it is part of a screening library. Looking further into this entry, we realized we are missing detailed annotation for this compound and started to investigate broader from more sources for this entry point. As suramin has many different targets (72 potential targets according to Wiedemar et al. (2020) it seems at first glance like a good candidate for initial screening, yet Bachmann et al. published already in 1999 (Bachmann et al., 1999), the “*potent inhibition of the CFTR chloride channel by suramin*.” Thus, merging of available resources can help to plan experiments and interpret results by using additional annotations, in this case to extend the annotation of suramin to CFTR inhibitor.

Substances can activate CFTR at several steps during its maturation pathway from a nascent polypeptide chain to a fully functional membrane protein (Lukacs and Verkman, 2012; Veit et al., 2016): CFTR mRNA is transcribed in the nucleus and translated into a polypeptide chain in the endoplasmatic reticulum. If misfolded, CFTR is recognized by the ER quality control, and the protein is degraded by the proteasome (ER-associated degradation, ERAD pathway). Correctly folded CFTR is promoted to the Golgi apparatus by the ER-associated folding pathway ERAF. In the Golgi apparatus, CFTR is complex glycosylated by MGAT enzymes and transported via vesicles to the apical membrane. In the subapical compartment, CFTR can be degraded via lysosomes (LY). Only 30% of wild-type CFTR are processed to the apical membrane and many CF-causing mutations such as F508del undergo much lower maturation rates (Lukacs and Verkman, 2012). Thus, we sought to annotate the substances according to three distinct functional categories: according to their influence on CFTR (active/inactive/inhibitory), their order of interaction (direct = binds to CFTR, indirect = influences a pathway that is important for CFTR), and the cellular compartment in which the substance causes the CFTR-relevant effect (nucleus, ER and Golgi compartment, apical membrane, and subapical compartment) (Table 1). The functional categories have been retrieved from the assessment of the authors and concatenated for all publications that report on one substance. This sum of author-guided statements, retrieved by conventional text mining, on how the substance acts on CFTR has next been used to assign an entry in each category to the substance.

**TABLE 1:** Classification of CandActCFTR substances in 2019/2010

Category: influence on CFTR function	
Enhances CFTR function	354
Likely enhances CFTR function	31
Inhibits CFTR function	14
None	217
Inconsistent assignment	18
Unknown	2,441
Category: order of interaction	
Binds to CFTR	83
Binds to an interaction partner of CFTR	1
Indirect	68
Unknown	2,923
Category: subcellular compartment	
Apical membrane & subapical compartment	88
ER & Golgi (translation, quality control, trafficking, PTM^a^)	50
Nucleus (transcription)	8
More than one compartment	71
Unknown	2,858

a Post-translational modification, e.g., glycosylation.

We next have analyzed the data set annotated for influence on CFTR function, order of interaction, and subcellular compartment by principal component analysis (Figure 4). Compounds that enhance CFTR function occupy the central space of the PC diagram whereby their occurrence roughly reflects the density of compounds tested. Three structural outliers were seen, indicating an area of interest in the chemical space in a region, where few tested compounds are within our database, which are fusicoccin (Stevers et al., 2016), the non-hydrolyzable ATP analogon B44 (Miki et al., 2010), and NSC-11237 (Odolczyk et al., 2013). When stratified by subcellular compartment, we could observe two clusters of substances that act at the apical membrane in the subapical compartment. However, some CFTR inhibitors and activators were located closely together, suggesting that the PC analysis needs to be adapted with respect to its dimensions if a segregation of such contrasting functionalities in distinct clusters has to be achieved. In summary, CandActCFTR collects data from different resources such as 12 systematic screens for compounds undertaken for cystic fibrosis in academia (Galietta et al., 2001; Yang et al., 2003; Pedemonte et al., 2005; Carlile et al., 2007; Robert et al., 2008; Xu et al., 2008; Cateni et al., 2009; Pedemonte et al., 2011; Phuan et al., 2011; Carlile et al., 2012; Namkung et al., 2013; Cui et al., 2016). Through the joint analysis of these data, the chemical space used by the compounds becomes accessible and can be employed for compound selection: when chemical structures of the tested compounds are displayed in a principal component analysis in their chemical space, structural similarities between compounds that share annotated features such as “mode of action” or “subcellular compartment” are visualized as clusters of substances in the chemical space. These clusters suggest a highly attractive area of the chemical space for substance optimization.

## Discussion

In the digital age where a huge amount of information is available, it is advantageous to organize such knowledge in a meta-database for retrieval and analysis of data. While in theory a good data structure design comes first, there is the issue of first knowing what the content of the meta-database is going to be (items and linkage between items). Under this condition, the entry forms can be designed first and the data can be directly entered into the structured database. In reality, one does not necessarily know in advance of starting screening publications for their information, which of the content is deemed to be interesting or needs be accumulated for analysis. To transform rather unstructured data into uniformly structured data, we looked at the process applied by the researcher using multiple screening and rescreening rounds to refine the data collection with each cycle. Many people rely on spreadsheets to organize this data. We identified a need regarding the handling of such a plethora of changing input tables, and joining the data from different tables with varying column titles, as well as scaling up from dozens to hundreds of publications. To tackle this goal of joining various sets of information extracted from literature texts, transforming them into tabular organized information excerpts, and to furthermore enable the organization of the content, we defined rules for these transformations. Our project can be used as an exemplary case on how to proceed when spreadsheets become the main entry mask format for a literature excerpt-based information organization and aggregation system. We propose using tools like the graphical workflow manager KNIME (Berthold et al., 2007), which can be taught to people untrained in IT processing within a very short time period to organize their data collection and ultimately clean their data, so that it is fit for analysis or distribution using web tools like a Grails (Grails Framework, 2021) server. Thus, we also provide our KNIME workflows accompanying the webserver, which can also be used independently.

We provide the software tool CandActCFTR, which can be repurposed for adaptation to other use cases and applications where chemical compounds with the structure, synonyms, InChIKey (Southan, 2013; InChI Trust, 2021), and literature are of interest and we provide our seed content dataset on CFTR-relevant substances (GitLab, 2021). CandActCFTR is a comprehensive research tool combining information on a growing amount of CFTR acting substances from different sources, mainly retrieved from publications in scientific journals, abstracts, and presentations on scientific meetings. CandActCFTR in its current form can be installed and operated at other sites. We have implemented a principal component analysis to visualize the similarity of substances and we have handled requests from the CF community to answer whether a certain substance is similar in structure to a CFTR activator listed in CandActCFTR (Figures 2 and 5).

Chemical properties of all substances in CandActCFTR have been assessed using principal component analyses (Figure 4) to identify those areas within the chemical space that are occupied by true-active substances. Other substance-related databases are available for medicinal and aromatic plant’s aroma molecules (Kumar et al., 2018), on therapeutic targets in *Campylobacter jejuni* (Hossain et al., 2018), a therapeutic targets database (Li et al., 2018b), and a database of structurally annotated therapeutic peptides (Singh et al., 2016). These are specialized databases like CandActCFTR, but among these examples, only CandActCFTR is provided as a generic tool that can be adapted by interested researchers to collect and analyze their data for other diseases and other therapeutic targets. Furthermore, the data set provided by CandActCFTR is, to the best of our knowledge, unique in that it compiles comprehensively the literature on CFTR activating substances and thus enables meta-analysis (Figure 4).

It was shown for CFTR-targeting cystic fibrosis molecular therapeutics that combination therapies are superior to approaches that build on single substances (Phuan et al., 2018; Southern et al., 2018; Veit et al., 2018), and thus our category definition considering subcellular compartmentation and the order of interaction will assist in selecting candidate therapeutics for that act on CFTR at several steps vital for CFTR gene expression, protein maturation, and activation. This phenomenon reflects that mutant CFTR is functionally deficient in several aspects (Veit et al., 2016): F508del is known as a processing mutant, failing to mature properly and at the same time, F508del-CFTR is functionally impaired if it reaches the apical membrane (Veit et al., 2016). To correct both properties of F508del-CFTR, the current therapeutic Orkambi combines a substance that promotes CFTR maturation and a substance that activates F508del-CFTR. Veit et al. (2018) and Phuan et al. (2018) have recently combined CFTR acting compounds to correct mutant CFTR whereby, interestingly, the individual substances had only a minor influence on CFTR (Veit et al., 2018). We have noticed that most substances in CandActCFTR could not yet be categorized (influence on CFTR—80% unknown, order of interaction—95% unknown, cellular compartment—93% unknown; Table 1) and envisage that future data will enable us to assign more substances to specific categories.

## Conclusion

CandActCFTR is a pilot project to merge data from publicly available sources and establish a database of candidate cystic fibrosis therapeutics for the activation of CFTR-mediated ion conductance. The acquired information on tested substances will assist in the identification of the most promising candidates for future therapeutics. Besides its specific application to identify CFTR therapeutics, we provide the software base of CandActCFTR as a tool for other chemoinformatics applications where properties of chemical molecules are at the core of interest; https://gitlab.gwdg.de/mnieter1/CandActBase. By not only providing a web service but also distributing the KNIME workflows used to prepare the loading of the data into our databases backbone, as well as the processing recipes for similarity searching, we hope to provide the tools for the community with the necessary flexibility to be of use in the future.

## Data Availability

The raw data supporting the conclusions of this article will be made available by the authors, without undue reservation.
