# Supplementary material for: Comprehensive Analysis of Chemical Structures That Have Been Tested as CFTR Activating Substances in a Publicly Available Database CandActCFTR
Source: Front Pharmacol. 2021 Dec 8;12:689205. doi: 10.3389/fphar.2021.689205 (PMC8692862; doi:10.3389/fphar.2021.689205)
Supplement: Supplementary file 5 [file DataSheet1.docx]

Supplementary Material

# Supplementary Screen-**C**apture Data

Video capture demonstrations of CandActCFTR functionality; list of videos:

1. Exploring_PointCloud_TaggedByPaper_with_CompoundImagesAndAnnotationPopUps.mp4
2. Search_Ivacaftor_By_Name.mp4
3. Search_Ivacaftor_By_Drawing_Exact_Structure.mp4
4. Search_Ivacaftor_By_Drawing_Structure_One_MethylGroup_Off.mp4 i

**Supplementary Video 1.** Demonstrating the use of the point cloud depiction of the chemical space with a pop-up panel providing information, such as structure, and annotations

**Supplementary Video 2.** Demonstrates the use of search using the name search, looking for the compound Ivacaftor using the name

**Supplementary Video 3.** Demonstrates drawing a compounds graph using the chemical structure drawing plugin, resulting in a direct matching hit

**Supplementary Video 4.** Demonstrates drawing a compounds graph using the chemical structure drawing plugin, without resulting in an exact match, thus implicitly activating the similarity search routine for a single compound.
